# Case of Wound of the Throat, in a Maniac, in Which the Epiglottis Was Nearly Severed

**Published:** 1855-08

**Authors:** George Dock

**Affiliations:** Harrisburg, Pa.


*Case of Wound of the Throat, in a Maniac, in which the Epiglottis was nearly severed. Recovery.* By GEORGE DOCK, M. D., Harrisburg, Pa.

On the morning of the 30th of Nov. 1853, I was summoned to the Pennsylvania State Lunatic Hospital in great haste, one of the inmates, Mr. S——, having cut his throat whilst alone in the water closet adjoining his room. Upon entering his room I found a man, about sixty years of age, lying upon the floor, in a pool of blood, with his throat cut literally from ear to ear. Life was apparently extinct ; no pulse at the wrist, skin cold and bedewed with a death-like moisture ; eyes set and filmy ; teeth clenched and no respiratory movement of the chest. For a moment I supposed him dead. Placing my ear upon his naked chest, I however detected a faint pulsation of the heart and a feeble, smothered, respiratory whisper. I immediately had several of the attendants to apply vigorous friction to his extremities ; sinapisms were laid to the calves of his legs and to his arms, whilst I proceeded to ligate the—yet oozing—superior thyroidal and lingual arteries, from which, with the external jugulars, he had lost his blood, until from prostration he could bleed but little more.

Taking the opportunity of examining his wound more deliberately, I found he had cut between the thyroid cartilage and hyoid bone, severing the latter, with the tongue, from their attachments, the wound making a clean gap into the pharynx, about two inches wide: (the larynx being drawn down by the sterno-thyroid muscles that distance from the retracted hyoid bone.) I also discovered, after sponging out the clotted blood, that the epiglottis was cut, at its base, almost completely off; hanging but by a little cord on each side. To have thus left it would have been fatal. Of the expediency of putting a ligature into it, I, on the other hand, was somewhat in doubt. Concluding it to be the least of two evils, I armed a delicate, very sharp, curved needle with a fine silk thread, and proceeded in the following manner: I entered the needle at the centre of the base of the stump, only penetrating it about two-thirds of its thickness, then brought out the point of my needle on the top face of the wound, entered it on the corresponding face of the loose piece of the epiglottis, bringing the point of the needle out on its anterior face, about a line from the cut end, and drew the parts into neat and perfect apposition by tying the ligature, the ends of which I clipped close, trusting to the removal of them during the suppurative stage of the whole wound. Then came the larynx to be drawn up to its place and there retained. To accomplish this, I armed another needle with saddler's silk, pierced a fold of the cellular tissue and muscle on its sides—first one, then the other side; then carried the needle over a corresponding point of the hyoid bone, making it the tackle of my pulley, and with this loop I drew the root of the tongue, &c., and the larynx close together, tied my knot and cut off one end of each of these ligatures, bringing the other ends out with those from the arteries. Four interrupted sutures were then put into the integuments, and the wound was dressed in the ordinary manner. During all this time, my labors appeared to be spent upon a man in articulo mortis, yet still I determined to persevere.

I ordered a stiff, hot brandy toddy to be prepared and a stomach tube brought to me. But his jaws were so firmly set as to render it impossible to separate them by manual force; so I

took a large gum catheter and introduced it through his nose, as far down the œsophagus as it would reach, then took a small syringe, adjusted it to the catheter, and injected the toddy into him. This, in a short time, with warm, stimulating applications to his limbs, brought on reaction, which I *coaxed* on gently, feeling my way so as not to overdo the thing. I had the pleasure, before leaving him, to see him breathing nicely through the natural apertures, the pulse becoming quite perceptible at the wrists, and his surface restored to a moderate degree of warmth.

He gradually recovered, being fed on nutritious liquids by the tube; the wound granulated well, and in a couple of months he was sent home perfectly sound; his voice natural and strong, and his mind restored to a state of sanity.

*Remarks.*—The above case I think is one possessing several points of interest to the profession. The wound was an unusually extensive one; the hæmorrhage profuse, and his condition altogether unpromising. A wound of the epiglottis to such an extent is rare to recover from; and here let me call your attention to the fact, that, from the day of the injury until he recovered he had no spasmodic cough, and there took place perfect union in the epiglottis. You will also observe that in passing my ligature I did not penetrate the entire thickness of the epiglottis, so that the mucous membrane on the glottal face was left intact, to which circumstance, I think, was due, in a great degree, the absence of all irritation and cough. This was my object and idea at the time I did it, and if an erroneous one, I fortunately erred on the safe side.

To Dr. Curwen, the superintendent of the Hospital, and Dr. Dewitt, his assistant, much credit is due for their unceasing attention and kindly care to the patient during the progress of his convalescence.

